# Treatment of Acute Articular Rheumatism by Lemon Juice

**Published:** 1853-09

**Authors:** Hector Peltier

**Affiliations:** M. D. Edin., Professor of Institutes of Med., Montreal School of Medicine; Physician to the Hotel Dieu, &c.


*Treatment of Acute Articular Rheumatism by lemon juice.* By HECTOR PELTIER, M. D. Edin., Professor of Institutes of Med., Montreal School of Medicine; Physician to the Hotel Dieu, &c.—I wish to place before your readers the result obtained by myself with lemon juice in acute rheumatism. I have now used it since 1850, and whenever it could be properly tested. It is to Dr. G. O. Rees, of Guy's Hospital, London, that we are indebted for the employment of this medicine in rheumatism. I cannot give any reliable explanation of its decided benefits, because alkalies act also beneficially in many cases of a similar kind. The way I employed it was simply by directing that the lemon itself should be sucked by my patients now and then in the course of the day. They never used more than two per diem. Under its influence, the pain and stiffness of the joints diminished in four or five days and sometimes sooner.

The cases I have had were genuine acute rheumatism, with all the characteristic symptoms present, but free from complication with any inflammation of the heart. I have had frequent opportunities of testing what I would call this invaluable, though simple mode of treatment, in the Hotel Dieu of Montreal, as well as in private practice, for during the last winter and spring, rheumatism raged extensively in this city. I know well that all species of remedies have been tried in rheumatism, but none have given me so much satisfaction as lemon juice. As for colchicum, its reputation is losing ground; and I think with good reason. It is a powerful medicine, but has been used too freely, and given in altogether too large doses. From my experience, I am disposed to believe that the majority of practitioners will honestly confess that it has done more harm than real benefit in both gout and rheumatism, and as it has been lately remarked with great propriety by Dr. Bouchardat, in his Annuaires de Therapeutique for 1852, "the greatest number of cases of death from gout or rheumatism is more attributable to colchicum than to the metastasis of the disease, as we are prone to say when a bad result supervenes." Salines are very useful in acute rheumatism, and form the best purgatives that can be employed. I am in the habit of prescribing them once or twice a week in connection with the lemon juice throughout the duration of the case. Iodide of potassium I also use, but only in the latter state, as an alterative.

Dover's powder I have given at night to allay any irritation. Fomentations of the joints I order but seldom, and very sparingly, because they give more trouble than real advantage.

As an illustration of the preceding remarks, I will mention one case in particular that I treated in February 1853, which gave me a good deal of uneasiness on account of advice repeatedly offered to the patient by his surrounding friends. Mr. J. L——, a gentleman connected with the first banking institution in Canada, was affected with acute rheumatism. Every joint was immovable, the pain intense, skin hot and perspiring profusely, pulse 110. From past results with lemon juice, I decided to use it, and directed two lemons to be sucked daily, confining him to low diet, and barley water as a drink. Nothing else was ordered, but an occasional saline draught.

The improvement became sensible in less than a week afterward. During this period many friends called upon my patient, each one giving in his opinion, &c., all, I regret to say, against my mode of treatment. Being sure of a successful issue, from past experience, I paid no attention to their statements and insinuations, but continued my treatment, and to the amazement of all his friends, Mr. J. L—— returned to the bank the thirtieth day from the date of his illness, perfectly well, and has not suffered since, though the temperature has been very changeable during the spring and this summer.

If you think, Messrs. Editors, that the above remarks, although written currente calamo, are worth publishing, I shall thank you in advance, and give you my best wishes for your success.—*Montreal Journal of Medicine and Surgery.*

